# STatistically Assigned Response Criteria in Solid Tumors (STARCIST)

**DOI:** 10.1186/s40644-015-0042-4

**Published:** 2015-07-31

**Authors:** Thomas Bengtsson, Sandra M. Sanabria-Bohorquez, Timothy J. McCarthy, David S. Binns, Rodney J. Hicks, Alex J. de Crespigny

**Affiliations:** Biostatistics, Genentech Inc, 1 DNA Way, South San Francisco, CA 94080 USA; Clinical Imaging, Genentech Inc, South San Francisco, CA USA; Clinical Imaging, Pfizer Global R&D, Groton, CT USA; The Sir Peter MacCallum Department of Oncology, the University of Melbourne, Parkville, VIC Australia; Cancer Imaging, the Peter MacCallum Cancer Centre, East Melbourne, VIC Australia

**Keywords:** 18 F-FDG, FDG-PET, Drug response biomarkers, Tumor response, Signal detection, Threshold estimation, RECIST, PERCIST

## Abstract

**Background:**

Several reproducibility studies have established good test-retest reliability of FDG-PET in various oncology settings. However, these studies are based on relatively short inter-scan periods of 1–3 days while, in contrast, response assessments based on FDG-PET in early phase drug trials are typically made over an interval of 2–3 weeks during the first treatment cycle. With focus on longer, on-treatment scan intervals, we develop a data-driven approach to calculate baseline-specific cutoff values to determine patient-level changes in glucose uptake that are unlikely to be explained by random variability. Our method takes into account the statistical nature of natural fluctuations in SUV as well as potential bias effects.

**Methods:**

To assess variability in SUV over clinically relevant scan intervals for clinical trials, we analyzed baseline and follow-up FDG-PET scans with a median scan interval of 21 days from 53 advanced stage cancer patients enrolled in a Phase 1 trial. The 53 patients received a sub-pharmacologic drug dose and the trial data is treated as a ’test-retest’ data set. A simulation-based tool is presented which takes as input baseline lesion SUVmax values, the variance of spurious changes in SUVmax between scans, the desired Type I error rate, and outputs lesion and patient based cut-off values. Bias corrections are included to account for variations in tracer uptake time.

**Results:**

In the training data, changes in SUVmax follow an approximately zero-mean Gaussian distribution with constant variance across levels of the baseline measurements. Because of constant variance, the coefficient of variation is a decreasing function of the magnitude of baseline SUVmax. This finding is consistent with published results, but our data shows greater variability. Application of our method to NSCLC patients treated with erlotinib produces results distinct from those based on the EORTC criteria. Based on data presented here as well as previous repeatability studies, the proposed method has greater statistical power to detect a significant %-decrease on SUVmax compared to published criteria relying on symmetric thresholds.

**Conclusions:**

Defining patient-specific, baseline dependent cut-off values based on the (null) distribution of naturally occurring fluctuations in glucose uptake enable identification of statistically significant changes in SUVmax. For lower baseline values, the produced cutoff values are notably asymmetric with relatively large changes (e.g. >50 %) required for statistical significance. For use with prospectively defined endpoints, the developed method enables the use of one-armed trials to detect pharmacodynamic drug effects based on FDG-PET. The clinical importance of changes in SUVmax is likely to remain dependent on both tumor biology and the type of treatment.

## Background

FDG-PET is becoming increasingly important as a tool for assessing early treatment effects in clinical trials of novel oncology drugs [1]. To quantify FDG uptake in tumors, clinical trials often employ pre-defined visual scoring systems such as the Deauville criteria in lymphoma [2], Cheson, 2014 #79 or semi-quantitatively using standardized uptake values (SUVs) [3]. SUVs derived from static FDG-PET scans are used as a practical way to estimate regional glucose metabolism, and imaging protocol guidelines have been proposed to standardize how such scans are performed (e.g. [4]). A key outcome metric is the change in SUV during the treatment course and, for a given patient, this change is typically defined by the relative change from baseline in SUVmax averaged across a set of target lesions. Metabolic response assessments are commonly based on the widely used EORTC criteria [5], which define a partial metabolic response (or metabolic progression) as a decrease (increase) in target lesion SUV of >25 % compared to pretreatment baseline, but newer assessment methods have also been proposed [6, 7]. In the present work, we propose a novel, statistically assigned, metabolic response criteria (termed STARCIST) for use with serial FDG-PET. Our method is based on the detailed noise distribution derived from of test-retest data and accounts for multiple target lesions in a rigorous statistical manner.

Typically, phase-1 clinical trials of novel cancer therapeutics must establish the safety and maximum tolerated dose of the new drug. However it is increasingly desirable to demonstrate that the drug is at least engaging its target at this early phase of development and for this purpose FDG-PET is often used as a *pharmacodynamic readout* of drug activity. In this context, the aim is to detect a significant change in tumor FDG uptake (early in the treatment course) that can be ascribed to drug effects on glycolytic metabolism. We note that such effects are of interest during early drug development even though they may not ultimately result in cell death or subsequent tumor shrinkage (e.g. due to suboptimal dose, schedule, or target population). The EORTC criteria, or newer criteria such as PERCIST [6], are convenient to use but do not take into account the detailed noise characteristics of individual lesion SUV measurements and hence do not directly quantify the magnitude of naturally occurring changes in tumor FDG uptake. Therefore it is not clear whether EORTC or PERCIST can be appropriately applied when using FDG-PET as a pharmacodynamic readout of drug activity.

The need to define what constitutes a statistically significant effect on FDG-PET motivated an in-depth examination of the test-retest characteristics of serial tumor SUV measurements. Based on this examination, an algorithm is presented that generates baseline-specific confidence limits on the mean, relative change in tumor SUVmax under the assumption of no functional change over the measurement interval, i.e. under the (null) hypothesis of no actual drug effect or tumor progression. Our method requires knowledge of the nature of noise in clinical FDG-PET studies in oncology. Fortunately, as recently reviewed by de Langen et al. [7], several studies have established good test-retest reliability of FDG-PET in various oncology settings [8–12]. While these studies have generally shown that good reproducibility can be achieved over a short 1–3 day test-retest period, PET measurements in early phase trials are usually made over an interval of 2–3 weeks during the first cycle of therapy. In addition to PET instrumentation and procedural ‘noise’, this longer time interval of 2–3 weeks exposes the PET measurements to more biological variability (e.g. due to natural fluctuations in tumor metabolism and FDG plasma kinetics). Therefore, in constructing our algorithm, in addition to literature data on reproducibility, we have used a training FDG-PET dataset with a longer inter-scan interval of about 3 weeks, which is more clinically relevant in the context of early drug development.

## Methods

Our approach to derive confidence limits for the %-change in SUVmax from baseline involved several steps. Based on an available training data set (described in Sec 2.1), we estimated a distributional model (Sec 2.2) for the natural variability (i.e. noise) in relative change from baseline in SUVmax values for individual tumors. Once estimated, we used the model to create an algorithm (Sec 2.3) which produces 95 % confidence limits for spurious %-changes in SUVmax from baseline. The algorithm simulatenously accounts for all target lesions and can correct for certain biases (e.g. change in uptake time). We then discuss (Sec 2.4) factors that may affect the performance of the algorithm, including dependence on the noise standard deviation, and detail how the proposed method can be used to calculate p-values applicable to single lesions, single patients, and for an entire cohort. The Human Research Ethical Committees or the Institutional Review Boards of the institutions involved independently approved all clinical trials.

### The training data

In order to inform the noise parameters to be used in the STARCIST algorithm, we analyzed a ‘test-retest’ training dataset. This dataset consisted of serial PET/CT scans aquired across four study sites of 53 advanced-stage cancer patients with multiple, solid malignancies on the dose escalation stage of a phase 1 clinical trial of a novel drug. Based on other PD biomarkers and a lack of observed radiological responses, the dose levels in the training data were considered sub-pharmacologic and the drug was ultimately discontinued from development. Further, as shown (Sec 2.2), FDG-uptake was not altered (on the average) during treatment. We therefore consider these training data as a’test-retest’ data set, reflecting what might be expected in a phase I clinical trials of a drug that shows no net effect on glucose metabolism.

An imaging core laboratory prospectively qualified the scanners and all scans were acquired according to a pre-defined imaging charter. Although different scanner models were used across the 4 study sites, serial scanning was always performed on the same scanner for each patient. Audited imaging compliance parameters included FDG uptake time, administered activity, scanning direction and arm position, and pre-scan fasting blood glucose levels. Baseline scans were to be obtained within 14 days prior to treatment initiation, with follow-up scans targeted for day 14 after treatment. The mean separation of the two scans was 19.8 days (sd = 4.7). The target uptake time was 75 ± 10 mins; the observed mean difference in actual uptake times between the two scans was 0.2 mins (sd = 13.5). Scans were collected and centrally analyzed at the Peter MacCallum Cancer Centre with lesions confirmed by a single cancer imaging specialist (RJH) and with volume-of-interest measurements performed by a single reviewer (DSB) using an automated software package developed at the Peter MacCallum Cancer Centre (MARVn). This software has been validated against a number of proprietary software packages available (manuscript in preparation).

SUVmax (corrected for weight) was measured in up to 6 lesions per patient (mean 3.8) for a total of 206 lesions located in the lung (*n* = 58), liver (*n* = 51), lymph nodes (*n* = 45), bone (*n* = 20), and other (*n* = 32). A linear mixed effects analysis showed that none of the imaging covariates (incl. monitored compliance parameters) were significant predictors of SUVmax at baseline or at follow-up (for details, see Appendix 1).

### Characterizing spurious changes in SUVmax

**Fig. 1:**
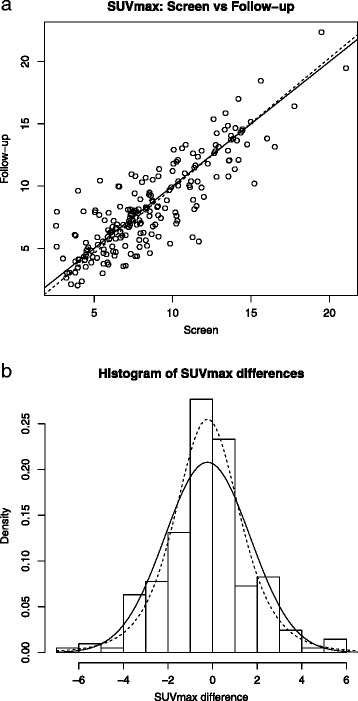
**a**) The scatterplot of SUVmax values highlights the strong correlation between baseline and follow-up values across lesions in our training dataset. The dashed regression line has a slope that is not significantly different from one. **b**) A histogram of the differences in lesion SUVmax between baseline and follow-up, which is approximated by a normal distribution of mean 0 and standard deviation 1.9 (*solid curve*). The *dashed curve* shows a t-distribution with 5° of freedom and scale parameter 1.49

In the training data set, as shown in the scatter plot in Fig. 1a, there was a strong correlation (*r* = .86) between SUVmax values at screening and follow-up, with most lesion values falling close to the line of identity (solid line). The estimated regression line in this plot (dotted) had a slope of 1.05 (SE = .038) and was not significantly different from unity (*p* = .24). A linear mixed effects analysis showed that the mean change in SUVmax did not vary signifcantly varied across trial sites (or scanners), tumor location, or with uptake time (t2-t1). (The details of these analyses are presented in Appendix 1.) Plotting the raw differences in SUVmax between the two time points for all lesions yields the histogram shown in Fig. 1b. As seen, although the data are slightly more peaked than the Gaussian distribution, the differences in SUVmax values are symmetrically distributed and are reasonably well approximated by a normal distribution with parameters set to the sample mean (−0.23, *p* < .16) and standard deviation (1.91) (solid curve). With parameters obtained by maximum likelihood (ML), this plot also shows a t-density with 5° of freedom and a scale parameter of 1.49 (dashed curve).

**Fig. 2:**
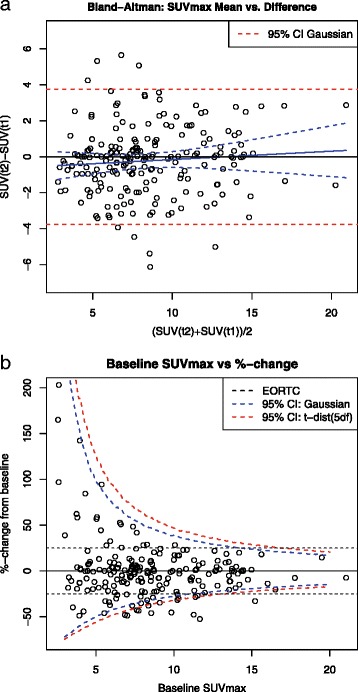
**a**) Change in lesion SUVmax in the training dataset plotted vs. the mean of the two measurements. The blue regression line has a slope that is not significantly different from zero. The *dashed blue lines* are 95 % confidence intervals on the regression line. Appr. 95 % of the changes in SUVmax are within +/− 4 units. **b**) Relative changes in SUVmax plotted vs. baseline SUVmax for each lesion. The *black dashed lines* show the ±25 % EORTC cut-off values, while the *blue* and *red dashed lines* show the confidence limits based on the Gaussian and t-distributions (5df), respectively

The histogram plot in Fig. 1b obscures the fact that there is a wide range of baseline SUVmax values among the measured lesions. A Bland-Altman plot, Fig. 2a, shows the same data on SUVmax changes as a function of the mean SUV value for the two timepoints. The main insight from Fig. 2a is that differences in SUVmax between the two measurements are essentially independent of mean SUVmax. Based on the preceeding analyses and plots, we note that the squared differences in SUVmax approximately follow a scaled chi-squared distribution. This distributional information enables us to evaluate the dependence of the *variance* of the SUVmax differences (i.e. 2*σ*^2^) on a set of covariates using a mixed effects Gamma regression. This regression showed no dependence of the variance of the change in SUVmax on trial site, baseline SUVmax, lesion location, diffrence in uptake time, or time between scans (cf., Appendix 1).

The preceeding analyses imply a simple, additive measurement model for SUVmax in which the observed value equals the ‘true’ SUV value plus a random zero-mean noise term with constant variance. That is, with *X*(*t*) representing the ‘true’ value of the SUVmax process at time t and *ε*(*t*) a zero-mean noise term with variance *σ*^2^, we observe *SUVmax*(*t*) = *X*(*t*) + *ε*(*t*). Then, under the (null) hypothesis of no change in the *X* -process from baseline (t1) to follow-up (t2), so that *X*(*t*2) = *X*(*t*1), the difference (*SUVmax*(*t*2) − *SUVmax*(*t*1) is mean zero with variance 2*σ*^2^.Fort our training data, the estimate of the per SUVmax observation noise standard deviation *σ* is therefore given by $\sigma =1.91/\sqrt{2}=1.36.$

In terms of response assessment, we are typically interested in relative changes in lesion SUVmax compared to baseline, as depicted in Fig. 2b. In this plot, the reciprocal operation implicit in the calculation of a relative change transforms the homoscedastic distribution of differences (Fig. 2a) in SUVmax into the asymetric and’funnel shaped’ pattern seen in the plot of the per lesion percent change. A key insight from Fig. 2b is that using fixed cut-off values at e.g. ±25 % severely underestimates the (true) test-retest variability in SUVmax for low SUV lesions, but overestimates this variation for lesions with high baseline values. Further, the 95 % confidence limits on natural variability in %-change in SUVmax when modeled by a normal distribution (blue curve, Fig. 2b) are markedly asymmetric about the origin, especially for low-avidity baseline lesions. This asymmetry derives from the reciprocal operation, and implies e.g. that a large %-increase in SUVmax is more likely to be spurious than a comparable %-decrease.

### The STARCIST algorithm

For a given patient, under the assumption of no systematic changes in the underlying SUV values across lesions, our algorithm produces percentiles for the distribution of spurious (noise-driven) relative changes in SUV between scans. By choosing appropriate lower- and upper percentile values from this null-distribution, we can create confidence limits for relative changes that are commensurate with the natural variability in measured SUV (e.g. choosing the 2.5 and 97.5 percentiles yields 95 % intervals.) Thus, if one observes a %-change in SUV that falls outside of this interval, the change is likely due to true increase in glucose metabolism or its reduction in response to therapy (depending on its sign), and the null-hypothesis of ‘no change’ would be rejected.

To calculate cut-offs (percentiles) for a given patient we proceed as follows. For each of *K* lesions, we simulate synthetic SUV observations at baseline and at follow-up and calculate the average %-change across lesions. This step is iterated a large number of times and the desired prediction interval is then based on the empirical percentiles of the simulated %-change across all iterations. Specifically, let *SUV*_1_, *SUV*_2_, …, *SUV*_*K*_ denote the values of *K baseline* SUV lesion measurements for a given patient, and, as suggested by the previously described additive measurement model for observed SUV-values, calculate for *j* = 1, …, *K*,.

$$ SUV{(baseline)}_j={SUV}_j+{\varepsilon}_{1,j} $$

$$ SUV{(followup)}_j={SUV}_j+{\varepsilon}_{2,j} $$

In the above, the noise terms *ε*_1,*j*_; *ε*_2,*j*_(*j* = 1, …, *K*) are sampled randomly from a zero-mean Gaussian distribution with standard deviation *σ*. Then, the average, (simulated) relative change in SUV is computed as

$$ \overline{r}={K}^{-1}{\displaystyle \sum_{j=1}^K}\frac{SUV{\left( follow\  up\right)}_j}{SUV{(baseline)}_j}-1. $$

For a given patient this metric is repeatedly iterated a large number of times (at least 10,000), each time using different random samples from the noise distribution. Empirical percentiles are then calculated from the resulting sampling distribution of $\overline{r}$. In the case of a single target lesion, i.e. for *K* = 1, the thresholds can be analytically calculated (see Appendix 3), but a closed form solution is not readily available when *K* > 1. As seen, in generating the null-distribution for $\overline{r}$, the STARCIST algorithm uses the observed baseline SUV values in place of the true (but unobserved) lesion SUV values. It can be shown that, for our setting, this replacement induces only a negligible (and ignorable) amount of bias in the estimated percentiles (e.g., the 2.5 and 97.5 percentiles).

However, care must be taken to ensure that the simulated values *SUV*(*baseline*) and *SUV *(*follow up*) are greater than the background noise in the images. This is easily achieved by discarding sample draws *ε*_1,*j*_, *ε*_2,*j*_ which lead to a violation of the threshold rule, and results in draws from a truncated Gaussian distribution. In practice, this is also facilitated by avoiding inclusion of lesions with SUVmax values less than some predefined threshold, e.g. as defined by the PERCIST criteria [6].

Correlations between the sampled noise terms at each time point can be introduced to obtain simulated lesion values that parallel observed within-patient SUV associations, properly accounting for the effect of multiple lesions. For our training data, based on the preceeding mixed model analyses, the within-patient lesion SUVmax correlation is approxmately .3 to .5. The method also works for log-normal data, in which case the SUV values must be log-transformed before simulating thresholds (although for this case an analytic solution is readily available based on the properties of the Gaussian distribution).

### Extensions to STARCIST

A number of extenstions to the STARCIST algorithm can be applied to increase its accuracy and utility. The details for each are provided in Appendix 2.

#### Accounting for uncertainty in ***σ***

The cut-offs produced by the STARCIST algorithm are clearly dependent on the functional form of the noise distribution and its standard deviation ***σ***. In our training data we found ***σ*** = 1.36. Previously reported values are considerably smaller, but typically show considerable variability across studies: e.g., for SUVavg, Minn et al. [9] and Weber et al. [12] reports values of ***σ***=.64 and ***σ***=.32, respectively, while for SUVmax, Nahmias & Wahl (10) reports ***σ***=.81. To account for some uncertainty in the estimate of σ, the exact value used in each iteration of the simulation procedure can be drawn from a distribution of reasonable values for σ (including estimates from other studies) rather than using a fixed value. This approach is exemplified in the Results (Sec. 3.1).

#### Correction for bias due to changes in uptake time

We include the method of Beaulieu et al. [13] into our algorithm to correct for changes in uptake time between scans. The correction works by producing a shift in SUVmax that is (linearly) proportional to the change in uptake time and to the magnitude of the observed SUVmax value. In practice, the correction is applied within STARCIST by shifting the 95 % confidence limits (rather than modifying the observed SUVmax values).

#### Assigning p-values for changes in SUVmax at the single-arm trial level

Since STARCIST indicates whether the observed mean %-change for a given patient is statistically significant or not (e.g., with a Type I error rate of 0.05), and because patients are independent from each other, the total number of patients with significant changes in a study follows the binomial distribution. This fact allows us to estimate the overall probability at the trial level that a therapy has caused a significant change in SUVmax.

## Results

Our test data set consisted of 57 2nd/3rd line non-small cell lung cancer (NSCLC) patients receiving erlotinib. This cohort was the control arm of a global phase II study evaluating the novel drug MetMAb (study OAM4558g, [14]). FDG-PET data was acquired from 24 global sites at screening and at days 10–14 after starting treatment (median separation was 3 weeks, range 13–42 days). Good technical compliance was observed with the standardized image acquisition charter [15]. Images were collected and analyzed centrally by a commercial imaging contract research organization. All scans were measured by a single reviewer who recorded SUVmax values (corrected by weight) for up to 5 target lesions per patient with an overall focus on lesions that best represented a patient’s burden of disease. Target lesions were defined as most FDG avid with a size requirement of at least 15 mm in longest dimension and a measured SUVmax of at least two. A total of 157 lesions were recorded, primarily located in lung (*n* = 53), lymph nodes (*n* = 48), and liver (*n* = 30). Since correcting for differences in uptake time between the baseline and follow-up scans did not signifcantly alter the conclusions, we show only the analyses based on uptake time corrected SUVmax values.

### Waterfall plots of SUV based on STARCIST

**Fig. 3:**
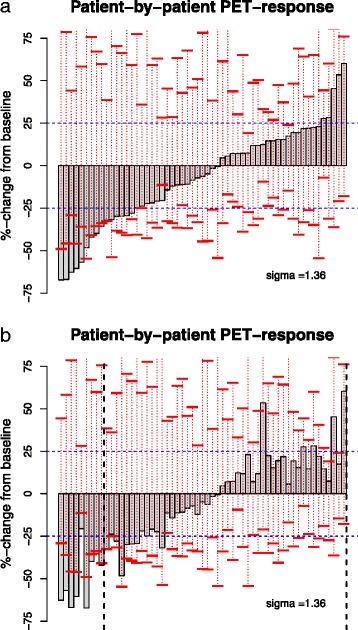
**a**) Waterfall plot of observed mean %-change in SUVmax for 60 NSCLC patients receiving erlotinib in the test dataset. The 95 % limits for spurious %-change in mean SUVmax based on our noise model are depicted by the vertical red lines. The fixed +/− 25 % EORTC cut-offs are given by the horizontal blue lines. Uptake time corrected changes in SUVmax are given by horizontal tick marks (*black*). **b**) Waterfall of same data as in **a**), but reordered according to the patient specific significance level (*p*-value) of the mean %-change in SUVmax. The smallest p-value for decreases in SUVmax is ordered from the *left*, with SUVmax increases similarly ordered from the *right*

For the 57 patients in the test dataset, as depicted in the waterfall plot in Fig. 3a, the primary PET review provided measurements for the mean %-change in SUVmax. Based on the +/−25 % EORCTC cut-off values, we obtain the distribution of metabolic responses shown in the top row of Table 1. Also shown in Fig. 3a, as produced by STARCIST with *σ* = 1.36, are baseline-specific 95 % intervals for spurious changes in SUVmax (overlaid on waterfall plot). Under the proposed criteria, if an observed %-change in SUV falls outside of its corresponding interval, we consider the null hypothesis of no significant change to be disproved and the patient to have demonstrated metabolic response or progression.

**Table 1:** Patient Level Metabolic Response Classification

Response classification	σ	PMR	SMD	PMD	*p*-value
**EORTC 25 %**	NA	15	37	5	< 2 × 10^− 12^
**STARCIST**	1.36	9	48	0	< 0.002
	0.81	15	39	3	< 2 × 10^− 10^
	1.00	13	44	0	< 4 × 10^− 6^

Patient level response classifications for the test dataset were based on the standard ±25% EORTC cut-off values and on the 95% confidence limits produced by STARCIST with σ set to 1.36 ,  0.81 and 1.0. The p-values are based on binomial distribution (n=57) based the total number of patients outside of the 95%-confidence limits. The p-value in the first row is derived by treating the EORTC criteria as 95% confidence limits

Waterfall plots rank patients in order of the size of observed change. An alternative approach is to order the plot according to the statistical significance (*p*-value) of the observed changes in SUVmax across patients, as shown in Fig. 3b. In this plot, the *p*-value for an individual patient is computed by considering the rank of the observed, mean %-change in SUVmax relative to the ranks of the corresponding, simulated %-changes produced by STARCIST. The plot is arranged so that the most significant decreases are ordered starting from the left while the most significant increases are ordered from the right. Figure 3b highlights that the most significant %-changes in SUVmax are often (but not always) also the largest %-changes.

Based on STARCIST, as shown in Table 1, 22.8 % (=13/57) patients differ in their response classification compared to those produced by the EORTC criteria. (These per-patient changes in response designation relative to EORTC are easiest to spot in Fig. 3b.) Because large, spurious %-increases in SUVmax is somewhat common, especially for patients with one or more faint baseline lesions, no patients in this analysis (with *σ* = 1.36) exhibit a statistically significant increase in SUVmax. In particular, the two patients with the largest mean, %-increases in SUVmax both have at least one baseline lesion with SUVmax value below 4. As seen (Table 1), a smaller noise standard deviation *σ*=.81 produces more metabolic responders and progressors compared to *σ* = 1.36. In this case, approximately 14 % (=8/57) of patients differ in their response classification based on STARCIST relative to those based on EORTC criteria.

Accounting for unertainty in σ  As mentioned (Sec 2.4), to account for the fact that the noise standard deviation is only approximately known, one can jointly sample values of *σ*^2^ and the error terms *ε*_*i*,*j*_ (*i* = 1, 2, *j* = 1, …, *K*) at each interation in STARCIST (for a given patient). To illustrate this idea, we sample *σ*^2^ from the inverse Gamma distribution with shape and scale parameters both set to 15. This distribution for *σ*^2^ was chosen to represent a compromise between *σ*=.81 (as reported by Nahmias & Wahl (10)) and *σ* = 1.36 (as in our training data). The resulting draws of *σ*^2^ are centered at 1, and are such that the 2.5 and 97.5 %-quantiles for *σ* (i.e., the noise standard deviation) are approximately located at .80 and 1.36, respectively. By allowing for a set of plausible values of the noise standard deviation, the metabolic response classifications based on this approach (presented in last row of Table 1), represent a sort of average of the previously considered values of *σ*.

Table 1 also gives the *p*-values associated with the total number of patients whose mean, %-change in SUVmax fell outside of the 95 % confidence limits and, for *σ* = 1.36, the signifcance level is approxmately .002. For *σ*=.81, the corresponding *p*-value is approximately 10^− 10^, a level of signifcance which may indicate that the error standard deviation *σ* is set too low. If one similarly treats the EORTC +/−25 % cut-offs as 95 % intervals the trial significance level is of the order 10^− 12^.

### Case examples

**Fig. 4:**
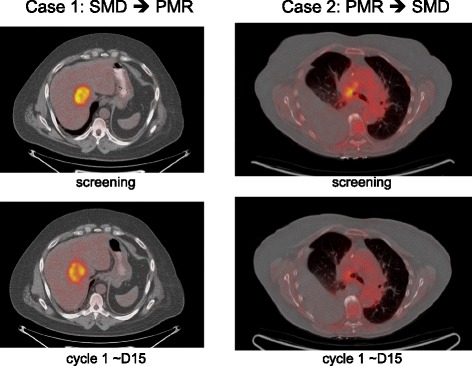
Example fused axial PET/CT images for patients with metastatic NSCLC whose response classification was changed using our new criteria (compared to the EORTC criteria). Case 1 had a single liver lesion that showed a 20 % decrease in SUVmax after 2 weeks of treatment and was classified as SMD by EORTC criteria. This patient was reclassified by our scheme as PMR because the baseline lesion SUVmax was 19, resulting in a narrow confidence interval for spurious changes. Case 2 has a single upper lung lesion demonstrating a 33 % decrease in SUVmax that was classified as PMR using the EORTC criteria. This patient was reclassified to SMD using our criteria, as a result of a low screening SUV value of 3.3. (These cases are represented in Fig. 3b as patients #4 and #22)

To illustrate cases where the response designation is driven by high- or low-intensity lesions, Fig. 4 shows PET/CT images from two patients falling into these categories. In the first case, a well defined and clearly FDG avid liver metastasis (the most avid lesion in this patient) visually decreased in intensity after 2 weeks of treatment and demonstrated a 20 % decrease in SUVmax. This patient was classified SMD by the EORTC criteria but PMR by our new criteria because the liver lesion had a baseline SUVmax of 19, resulting in narrow confidence limits for spurious change (−11.5 to 28.7 %). The second case example had a single lesion in the upper right lung that visually decreased in intensity after 2 weeks of therapy, and was classified by EORTC criteria as PMR with a 33 % decrease in SUVmax. However, the baseline SUXmax for this lesion was only 3.3, resulting in wide confidence limits for spurious change (−54 to 119 %), which encompass the observed % change and leads to reclassification as SMD. These two cases can also be seen in Fig. 3b as patients #4 and #22 (counting from the left), respectively.

## Discussion

Our observation of baseline-dependent fluctuations in SUVmax in the training dataset is consistent with a recent retrospective analysis of several previous test-retest datasets by de Langen et al. [7]. Their analysis also demonstrated the decreasing size of the test-retest %-change in SUV metrics with increasing baseline tumor SUV, a point also discussed by Weber et al. [12] (cf. their Figs 2 and 3). Similarly to our training data, Weber et al. [12] also substantiated the normal distribution for spurious changes in SUV.

Although our conclusions regarding the distribution of SUVmax test-retest data are similar to those of de Langen et al. [7], our method to define *statistically significant changes* in SUVmax differs from their approach in the way that thresholds (critical values) are defined, i.e., in the way the Type I error rate is controlled. To address the noisy nature of dim lesions (at baseline), de Langen et al. [7] propose cut-off regions that depend both on minimum absolute %-change in SUVmax *and* a concomitant minimum absolute change in SUVmax (cf., their Table 2). We note here that is not mathematically (or statistically) necessary to simultaneously consider both percent- and absolute changes, as claimed by de Langen et al. [7] (cf. their Results). In contrast, thresholds produced by STARCIST define cut-offs for spurious %-changes in SUVmax that vary continuously with baseline SUV, but do *not* require simultaneous consideration of absolute changes.

**Table 2:** Lesion Level Metabolic response Classification

		de Langen et al. (2012)		
Thresholds by STARCIST		PMR	SMD	PMD
	PMR	27	13	0
	SMD	0	98	8
	PMD	0	6	5

Lesion level metabolic response classifications for the test dataset based on STARCIST and the thresholds for SUVmax provided by de Langen et al. (cf. Table 2.1 in (7))

Our approach accounts for the fact that the spurious variation in %-change in SUVmax is highly asymmetric about zero. Such asymmetry requires that, e.g., a 40 % increase in SUVmax be treated *differently* than a 40 % decrease; i.e., on the %-scale, increases and decreases should have different cut-offs. In contrast, thresholds that include an absolute %-scale have to guard against large, spurious %-increases, and will consequently yield less power in detecting a drug effect that suppresses glycolytic metabolism, a fact that is illustrated in Appendix 3. This is especially true for low SUV lesions, which are likely to produce larger spurious increases than decreases (Fig. 2b). Additionally, by seamlessly accounting for differing numbers of target lesions, STARCIST produces a well-defined patient level response metric. Finally, our approach also allows for control of various sources of systematic bias. As an example, we have included a correction for variations in tracer uptake time. Although not currently included, correction for changing blood glucose levels [16, 17] and variations in plasma FDG kinetics [18] are easily incorporated.

We demonstrated our approach using SUVmax, yet STARCIST is equally applicable to SUVpeak, SUVaverage (normalized by weight or lean body mass), total lesion glycolysis, or any other metric for which appropriate test-retest (i.e. training) data is available. For instance, thresholds for the PERCIST criteria [6] can easily be computed using the presented simulation algorithm by calculating the %-change in SUVpeak from hottest baseline- to hottest follow-up lesion, although some added complexity is needed to account for the uptake in newly detected lesions. Unfortunately, since our test clinical trial dataset did not include SUVpeak measurements we were unable to directly compare STARCIST with PERCIST. Similarly, with the approach of de Langen et al. [7], it is not straightforward to process multiple target lesions per patient (personal communication, de Langen, 2014); indeed the issue of whether to focus on multiple target lesions or a single (hottest) lesion remains a matter of debate. Nevertheless, we have shown that we do obtain *different* results (altered metabolic responses in ~25 % of patients) compared to the simpler but still widely used EORTC criteria. The reader may reasonably ask which of the above referenced methods produces the ‘best’ results, but further work will be required to fully establish the performance of STARCIST relative to other approaches. Here, with focus on relative changes from baseline, we claim only that our method produces thresholds with the correct Type I error rate.

Regardless of the SUV metric employed, when using STARCIST the assumption is made that the noise function and variance (*σ*^2^) is relevant for the dataset being processed. Of course, an analogous assumption must be made when applying the PERCIST or EORTC criteria prospectively: namely, that the 25 % cut-off in SUVmax is valid for the scan conditions and tumor type (etc.) of the new study. At least, in the case of STARCIST, as noted, thresholds to determine metabolic response distribution are consistent with the noise distribution in SUVmax observations. We have also demonstrated that STARCIST can encorporate uncertainty in the estimate of the noise variance by drawing from a distribution of values of σ when simulating thresholds, instead of assuming a fixed value.

In considering the presented training dataset (Sec 2.1) as test-retest measurments, we assume no treatment effect and limited tumor growth over the 3 week scan interval scans. Of course, we cannot exclude the possibility that some patients may actually have a degree of tumor progression balanced by a weak drug effect, resulting in zero net change in FDG uptake. However, the lack of change in other PD biomarkers in this study and lack of clinical benefit suggests that this scenario does not dominate our data. Our estimate of σ (1.36) is larger than some earlier works, but this value is based on serial SUVmax measurements separated by a clinically relevant interval of approximately 3 weeks. In comparison, test-retest measurements made over a 2–3 day interval would certainly capture variability from technical sources, such as scanner hardware, reconstruction related biases and partial volume effects [19], as well as ‘procedural’ factors such as changes in FDG uptake time, injected dose, and blood glucose. However, over a longer time interval of ~3 weeks, additional biological sources of variability are likely to affect the measurements, e.g. variation in tumor (and indeed whole body) FDG metabolism and plasma FDG kinetics, increasing spurious changes between observations.

We believe that STARCIST will be a useful analytical tool for the pharmaceutical industry and researchers involved in drug development seeking to use FDG-PET as a PD biomarker of biological response. Based on our best estimate of the noise distribution in SUVmax measurements, the proposed methodology is rigorously rooted in classical statistical testing theory (e.g. see Chapter 5 of [20]) and produces baseline-dependent thresholds on the %-scale for an arbitrarily chosen significance level. We note, however, that metabolic response on the primary lesions will not necessarily translate into subsequent morphological response or, more importantly, clinical benefit. Indeed, for patients with substantial heterogeneity of response across lesions (including newly detected lesions), the worst performing lesion may drive the clinical outcome. Nonetheless, it is very likely that patients with and without statistically significant changes in SUVmax will have differing probabilities of clinical response.

Our method is relatively complex and is best implemented by means of a web interface so that a user can upload a table of SUV values, define a set of assumptions such as noise variance, and receive back a set of metabolic response designations for each patient. At the time of this printing, the STARCIST algorithm can be used at www.starcist.org.

## Conclusions

Test-retest data with a 3-week interscan interval demonstrated greater noise variance than published data with a short (1–3 days) time between measurements. Using our knowledge of the noise distribution in SUVmax, we propose a data driven approach to defining patient-specific cut-off values to determine statistically significant changes in tumor FDG uptake. Our approach takes into account multiple lesions per-patient and allows for systematic biases such as variation in tracer uptake time. Importantly, baseline tumor SUV strongly determines the variability in %-change in SUV over time: less intense lesions at baseline show greater spurious %-change. Additionally, the statistical framework also allows testing for significance at the level of the whole trial, even for a single-arm study. The technique is applicable to other lesion SUV measurements (such as SUVpeak) but suitable test-retest FDG-PET data, ideally with a 2–3 week interscan interval, is needed for the metric and preferably also disease of interest. We have shown that our methodology can change the metabolic response classification in more than a quarter of subjects in a phase 2 clinical trial of NSCLC patients.

## Appendix 1: Evaluating the effects of covariates on SUVmax in the training dataset

*Methods*

The linear mixed effects model and the generalized linear model were used to evaluate any dependence of mean SUVmax, and the dependence of mean and variance changes in SUVmax, on a set of covariates (e.g. uptake time and lesion location). All regression analyses were performed at the lesions level and treated study sites and patients (nested within sites) as mixed effects, and were done in the statistical package R [21] using the lmer, glm, and glmer functions. All reported p-values are two-sided and are based on model output.

*Mean SUVmax*

A linear mixed effects analysis showed that neither lesion location (baseline: *p* = .18; follow-up: *p* = .08) nor uptake time (baseline: *p* = .39; follow-up: *p* = .84) were significant predictors of SUVmax. At baseline, the variance components estimates obtained from this analysis were 0 for study sites, 3.3 for patients (nested within sites), and 9.0 for residuals. At follow-up, the same figures were 0, 4.8, and 8.5, respectively. Since all patients were scanned on the same scanner within sites, there was no effect on SUVmax due to scanners employed.

*Mean change in SUVmax*

A linear mixed effects analysis was used to assess if the mean change in SUVmax varied across trial sites, tumor location, or with uptake time (t2-t1). The estimated variance components for trial sites, patients, and residuals were 0, 1.60, and 2.05, respectively, indicating that study site did not play a significant role for changes in SUVmax. Further, there were no significant effects on change in SUVmax due to tumor location (*p* = 0.11) or uptake time (*p* = 0.29).

*Variance of change in SUVmax*

A mixed effects Gamma regression showed no dependence of the variance of changes in SUVmax (i.e. 2*σ*^2^) on baseline SUVmax (*p* = .54), difference in uptake time (*p* = .84), number of days between scans (*p* = .40), or lesion location (*p* = .10). Further, in this analysis, the variance components for trial sites and patients were both estimated to be zero, indicating a lack of dependence on the variance of changes in SUVmax on these factors.

## Appendix 2: Extensions to STARCIST

*Accounting for uncertainty in****σ***

Simulations under different distributional models for noise in SUVmax show that it is the reciprocal operation involved in calculating the relative change, rather than the exact form of the distribution, that drives the magnitude of the estimated percentiles. Thus, for practical purposes, we believe that the Gaussian (or t-) distribution suffices to provide informative results. More importantly, a reasonable estimate for *σ* is required, and in our training data we find ***σ*** = 1.36. Previously reported estimates of this parameter vary across test-retest studies. To account for the uncertainty in the noise standard deviation, one can generate samples from the joint distribution of ***ε***_1,***j***_, ***ε***_2,***j***_(***j*** = 1, …, ***K***) and ***σ*** at each interation in the algorithm using draws from the commonly employed Normal-Inverse Gamma distribution e.g. [22]. Specifically, to augment STARCIST, one first draws ***σ***^2^ from the Inverse-Gamma distribution, and (conditionally on ***σ***^2^) one draws noise terms ***ε***_1,***j***_, ***ε***_2,***j***_(***j*** = 1, …, ***K***) with variance equal to the sampled value of ***σ***^2^. The parameters relating to the Inverse-Gamma part of the joint density can be informed by previously reported estimates, or simply chosen so that a reasonable range of ***σ*** –values are represented by the algorithm.

*Correction for bias due to changes in uptake time.*

We include the method of Beaulieu et al. [13] into our algorithm to correct for changes in uptake time between scans. With this approach, we approximate what the SUV value at follow-up would have been had it been measured at the baseline uptake time. Specifically, with *β*(*SUV*(*t*2)) > 0 a correction factor derived from Beaulieu et al. (13), SUV (t2) is extrapolated linearly from the follow-up uptake time (t2) to the baseline uptake time (t1) using the correction *SUV*_*corr*_ = *SUV*(*t*2) + *β*(*SUV*(*t*2))(*t*1 − *t*2). For a given patient, the uptake time correction can be included in the STARCIST algorithm when SUV-values are sampled at follow-up by simulating *SUV*(*follow up*) = *SUV*_*j*_ + *δ*_*j*_ + *ε*_2,*j*_, where *δ*_*j*_ is the bias correction on the j:th lesion at t2. The estimation of confidence limits and systematic bias into a single algorithm is implemented in the R. The inputs to the algorithm are the baseline and follow-up SUVmax values for all lesions measured in a patient, the uptake times t1 and t2, and the standard deviation *σ* of the noise distribution.

*Assigning p-values for changes in SUVmax at the single-arm trial level*

The construction of cut-offs based on the null distribution of relative changes in SUVmax enables the use of single arm clinical trials to detect a pharmacodynamic drug effect on FDG uptake. Specifically, under the assumption of no underlying change in apparent lesion uptake between scans, with a specified type 1 error rate of *α* and a sample size of N, we expect about *N* × *α* of the treated patients to have mean %-changes in SUVmax which fall outside of their respective 100 × (1 − *α*) % prediction limits. Moreover, since patients are independent, the total number of mean %-changes outside of their respective confidence limits, here denoted S, follows the binomial distribution with parameters N and *α*. A p-value associated with a particular trial outcome is then given by $$ \overline{F}\left(S-1;N,\alpha \right) $$, the survival function for the binomial distribution evaluated at *S* − 1 ‘successes’. To focus this metric on metabolic responders only, we simply divide the type 1 error rate in half and calculate the p-value as $$ \overline{F}\left({S}_{PMR}-1;N,\raisebox{1ex}{$\alpha $}\!\left/ \!\raisebox{-1ex}{$2$}\right.\right) $$, where *S*_*PMR*_ denotes the total number of patients that fall below the lower prediction limits. We note that the p-values discussed here pertain to the outcome of the (entire) trial, i.e. to the clinical endpoint *S* or *S*_*PMR*_.

## Appendix 3: Lesion-by-lesion response classification

For the 57 patients in test data (Sec. 3), Table 2 presents a lesion-by-lesion comparison of metabolic response classifications based on STARCIST with the classifications based on Table 2.1 of de Langen (7). As seen, there is large degree of agreement between the two methods; in particular, 130 lesions (out of 157) are designated as having the same metabolic response classification. However, STARCIST classifies 13 more lesions as PMR (40 vs. 27). We outline below the theoretical reasons why our method produces a greater number of PMR classified lesions.

When the spurious differences in SUV between serial scans follow a symmetric distribution as, e.g., seen in our training data, and as reported by Weber el al. [12], then the thresholds of %-decreases vs. and %-increases can be highly asymmetric. That is, for the data and model considered in Sec. 2, under the null hypothesis of no true change between scans so that *μ*_1_ = *μ*_2_ = *μ*, the probability of getting a spurious change in the ratio below some threshold *k* is given by $$ p\left(\frac{Y2}{Y1}<k\right) = p\left(Y2-k*Y1<0\right)=p\left(Z < \frac{-\left(1-k\right)\mu }{\sigma_{\varepsilon}\left(1+{k}^2\right)}\right), $$where *Z* is drawn from the standard normal distribution. Equating the term − (1 − *k*)*μ*/*σ*_*ε*_(1 + *k*^2^) to (say) the 2.5 %-quantile of the standard normal distribution, i.e. to *Z*_.025_ = − 1.96, and solving the resulting quadratic in *k* yields the lower (and upper) thresholds for relative changes with a Type I error rate of 5 %. Specifically, with $$ CV=\raisebox{1ex}{${\sigma}_{\varepsilon }$}\!\left/ \!\raisebox{-1ex}{$\mu $}\right., $$ the threshold *k*_.025_ = $$ \frac{1 - \sqrt{1-{\left(1-{Z}_{.025}^2*C{V}^2\right)}^2}}{\left(1-{Z}_{.025}^2*C{V}^2\right)} $$ is such that $$ p\left(\frac{Y2}{Y1}<{k}_{.025}\right)=.025. $$ By symmetry, the upper threshold *k*_.975_ is given by the second root of the preceding quadratic. Simple algebra can then be used to verify the asymmetry in the (null) distribution of the sample ratio Y2/Y1, as reflected by the resulting inequality |1 − *k*_.025_| < |*k*_.975_ − 1|. With obvious changes to the sample quantiles, the preceding arguments hold for other Type I error rates (e.g., 0.1, or 0.01), and also to other symmetric distributions (e.g. the t-distribution.) We note: (1) the dependence of the thresholds on the mean (*μ*) and standard deviation (*σ*_*ε*_) through the coefficient of variation (CV); and, (2) that defining thresholds based on absolute relative changes results in thresholds which require larger %-decreases for statistical significance (but smaller %-increases.) Hence, relative to previously published methods, we expect our method to have higher power to detect (statistically significant) decreases in SUVmax, but also less power to detect increases.
